# Case report: Successful treatment of advanced pulmonary sarcomatoid carcinoma with BUBIB*-*ALK rearrangement and KRAS G12C mutation by sintilimab combined with anlotinib

**DOI:** 10.3389/fonc.2024.1269148

**Published:** 2024-02-13

**Authors:** Nana Huang, Tianhao Qu, Chunxia Zhang, Jia Li

**Affiliations:** Department of Oncology, The First Affiliated Hospital of Dalian Medical University, Dalian, China

**Keywords:** anlotinib, anti-angiogenesis, KRAS mutation, BUBIB-ALK rearrangement, PD-1inhibitor, pulmonary sarcomatoid carcinoma

## Abstract

Pulmonary sarcomatoid carcinoma (PSC) is a rare and aggressive subtype of non-small cell lung cancer (NSCLC) that is characterized by poor differentiation and invasiveness. According to the World Health Organization, PSC exhibits sarcoma or sarcomatoid differentiation and typically presents with an insidious onset, lacking specific symptoms and signs. It is associated with high malignancy, early metastasis, short survival time, and a poor prognosis. Treatment for PSC follows a similar approach to NSCLC; however, it presents significant challenges due to its high resistance to chemotherapy. Previous research has demonstrated the coexistence of two or more target mutations in PSC, and the presence of multiple mutations is correlated with higher mortality rates compared to single mutations. This is supported by our case study of a male patient with advanced BUBIB-ALK rearrangement and KRAS G12C missense mutation. There is currently no standard treatment protocol available for patients with this condition. The patient showed rapid progression after 1 month of alectinib treatment and was intolerant to paclitaxel + cisplatin chemotherapy. Following this, successful disease control was achieved with a combination therapy of sintilimab and anlotinib. The patient achieved a progression-free survival (PFS) of over 20 months, and long-term follow-up is still ongoing for the patient. Based on our clinical experience, the combination of anlotinib and programmed death-1 (PD-1) inhibitors may be a promising strategy for PSC patients, particularly those with multi-target mutations who do not respond to ALK-TKI and are resistant to chemotherapy.

## Introduction

Pulmonary sarcomatoid carcinoma (PSC) is a rare subtype of non-small cell lung cancer (NSCLC) characterized by mesodermal malignant tumor with sarcoma or sarcoma-like differentiation components. PSC originates from various tissues, including the lung parenchyma, bronchial and vascular walls, and bronchial cartilage (1). It accounts for approximately 1% of all NSCLC cases and represents only 0.1% to 0.4% of primary lung cancers (2). The World Health Organization (WHO) categorizes PSC into five distinct subtypes based on morphology: spindle cell carcinoma (SCC), giant cell carcinoma (GCC), pleomorphic carcinoma (PC), carcinosarcoma (CS), and pulmonary blastoma (PB) (3). PSC has the potential to occur at any age and is more frequently observed in males. Furthermore, certain studies have indicated a higher incidence of PSC among elderly men and heavy smokers (4).

The optimal treatment approach for PSC is currently a matter of debate, with the standard treatment typically following that of NSCLC. For patients with early-stage PSC, complete surgical resection is currently regarded as the most effective treatment option, which leads to improved survival rates. However, it is important to note that the rate of surgical resection is relatively low and carries a significant risk of postoperative recurrence (1, 5). Currently, there is no standardized protocol for the treatment of patients with advanced PSC. The current treatment approach primarily relies on the management of driver oncogene-negative NSCLC. In such cases, platinum-based combination chemotherapy is frequently used (6, 7). Due to its aggressive nature and high likelihood of frequent metastasis, PSC shows a poorer response to chemotherapy compared to other early-stage NSCLCs. PSC patients are prone to developing rapid drug resistance, resulting in tumor recurrence (8). The median PFS for PSC patients is typically only 2 months, with overall survival (OS) ranging from 4 to 6 months (9).

Notably, investigations have revealed a significantly elevated mutation rate of genes in PSC (10). Currently, the literature has documented several relevant genes, such as TP53, EGFR, KRAS, MET, and ALK, which can occur either independently or concurrently (11). The incidence of KRAS mutations was observed to be 30.6%, and patients with PSC who had KRAS mutations exhibited a poorer OS compared to those with wild-type KRAS (12). The incidence of ALK rearrangement in PSC among the East Asian population is noteworthy. In the Chinese population, the occurrence of ALK rearrangement in PSC is approximately 5%, which is consistent with other subtypes of NSCLC (13). ALK tyrosine kinase inhibitors (ALK-TKIs) have been extensively used in the treatment of patients with ALK-positive lung cancer, resulting in significant improvements in patient survival. The first-line therapeutic options for ALK-positive NSCLC include alectinib, brigatinib, ceritinib, crizotinib, and lorlatinib (14). While there are numerous reports on cases of ALK rearrangement currently available, the coexistence of ALK rearrangement and KRAS mutation is relatively uncommon (15). Furthermore, the majority of ALK fusion mutations involve EML4-ALK translocation (16, 17). Therefore, it is essential to conduct further stratified research and refine the management of ALK-positive patients in clinical practice.

This case presents the first documented successful treatment of advanced PSC patients with both KRAS G12C mutation and BUBIB-ALK rearrangement. The therapeutic approach employed a combination regimen consisting of sintilimab (a PD-1 inhibitor) and anlotinib. The patient demonstrated long-term stability, improved compliance, and absence of serious adverse reactions compared to chemotherapy.

## Case presentation

**Figure 1:**
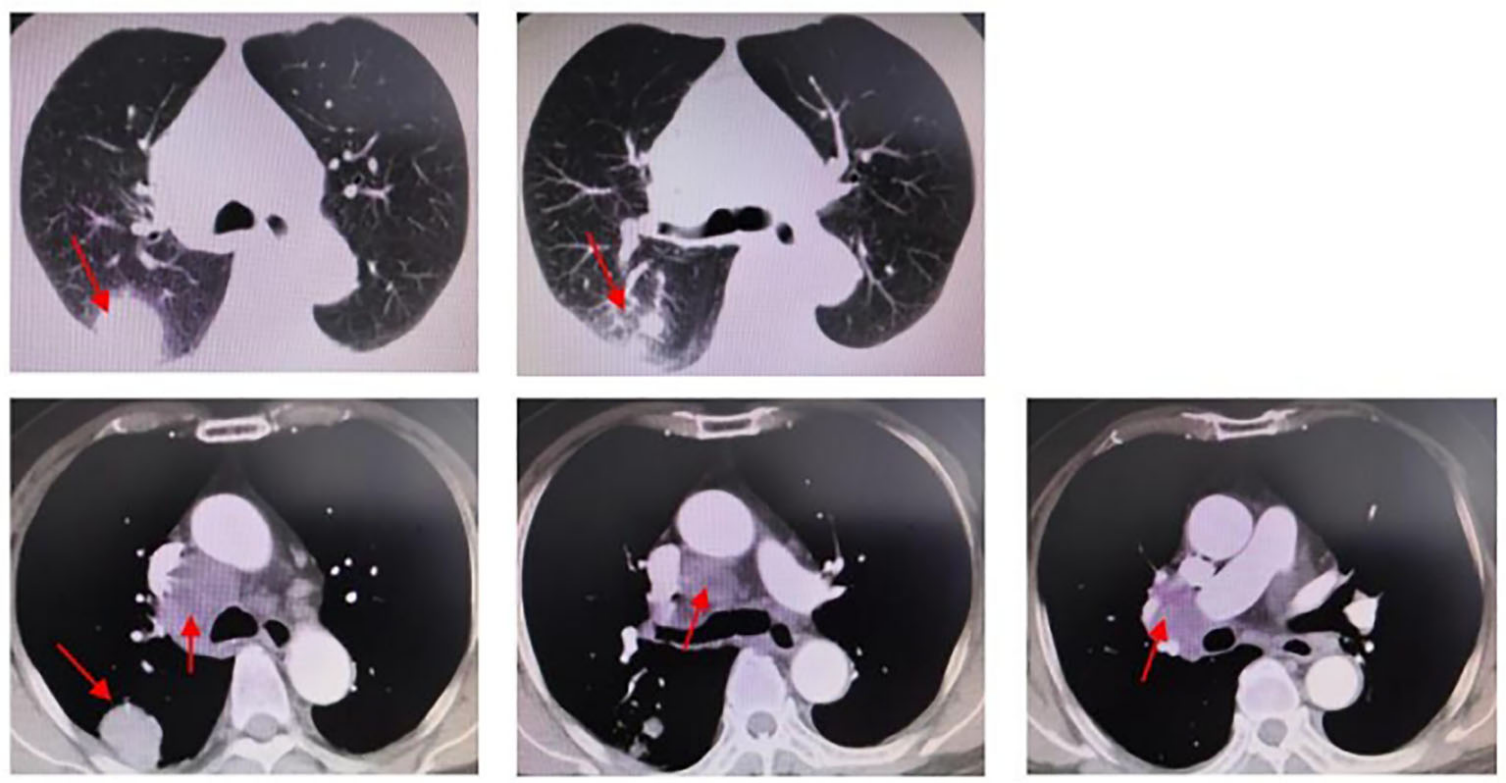
Chest CT (2020.12.18): peripheral lung cancer in the upper lobe of the right lung, approximately 3.39 cm in diameter. Right hilar and mediastinal lymph node metastasis; the larger one is approximately 4.47cm in diameter.

**Figure 2:**
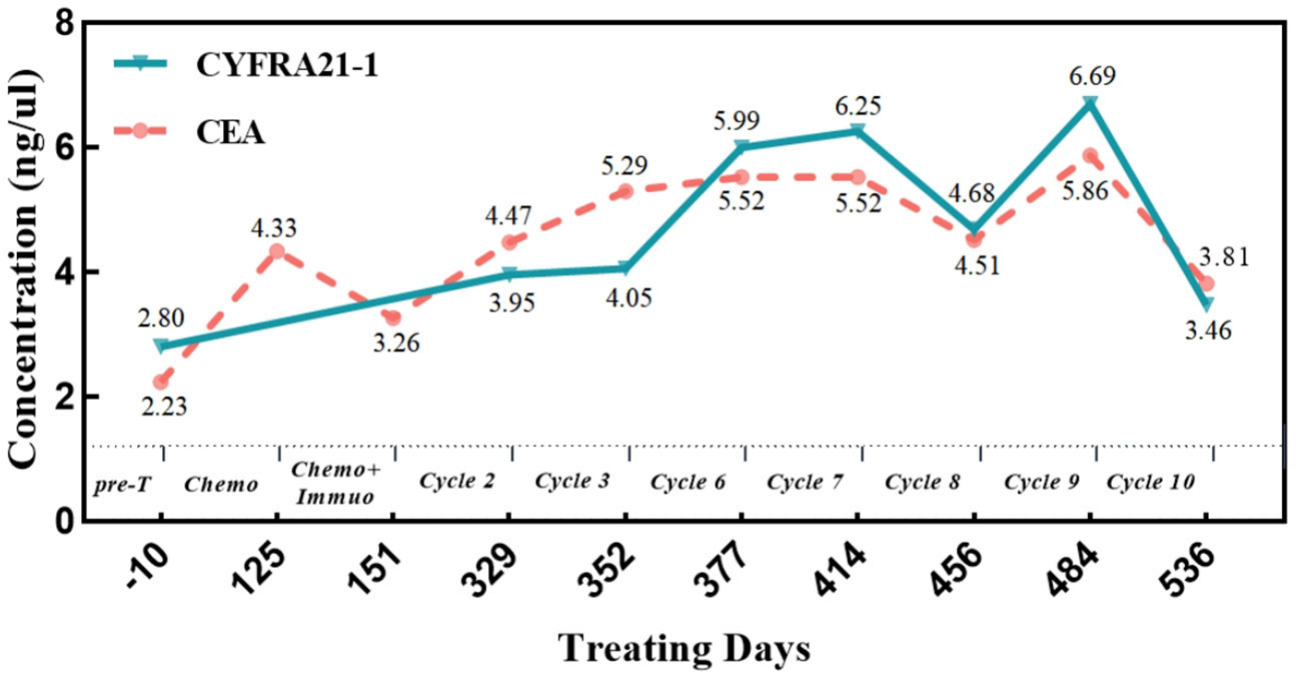
Changes of tumor markers during treatment. CYFRA21-1, cytokeratin 19 fragment; CEA, carcinoembryonic antigen; Pre-T, treatment program; Chemo, albumin paclitaxel + cisplatin chemotherapy; Chemo+Immuo, sintilimab + chemotherapy; Cycle 2/3/6/7/8/9/10, sintilimab + anlotinib treatment cycle.

In December 2020, a 78-year-old male patient with a 20-year history of diabetes, self-insulin control, and fasting blood glucose levels ranging from 10 mmol to 16 mmol presented at the hospital with complaints of weakness, cough, and persistent phlegm for 1 month. Additionally, he experienced persistent shortness of breath for 2 weeks. The patient had no family history of malignant tumors but had a smoking history of over 20 years. His ECOG score was 1. A chest computed tomography (CT) scan showed a cystic solid lesion measuring approximately 3.39 cm in diameter in the upper lobe of the right lung, along with metastases in the right hilar and mediastinal lymph nodes. The larger lymph node measured approximately 4.47 cm in diameter (**Figure 1**). No evidence of metastasis was observed on the upper abdominal plain CT scan and head CT scan. Laboratory examinations revealed normal results, with no abnormal increase in tumor markers. The level of cytokeratin 19 fragment (CYFRA21-1) was 2.80 ng/ml (reference range: 0–3.3 ng/ml), and the level of carcinoembryonic antigen (CEA) was 2.23 ng/ml (reference range: 0–5 ng/ml) (**Figure 2**).

**Figure 3:**
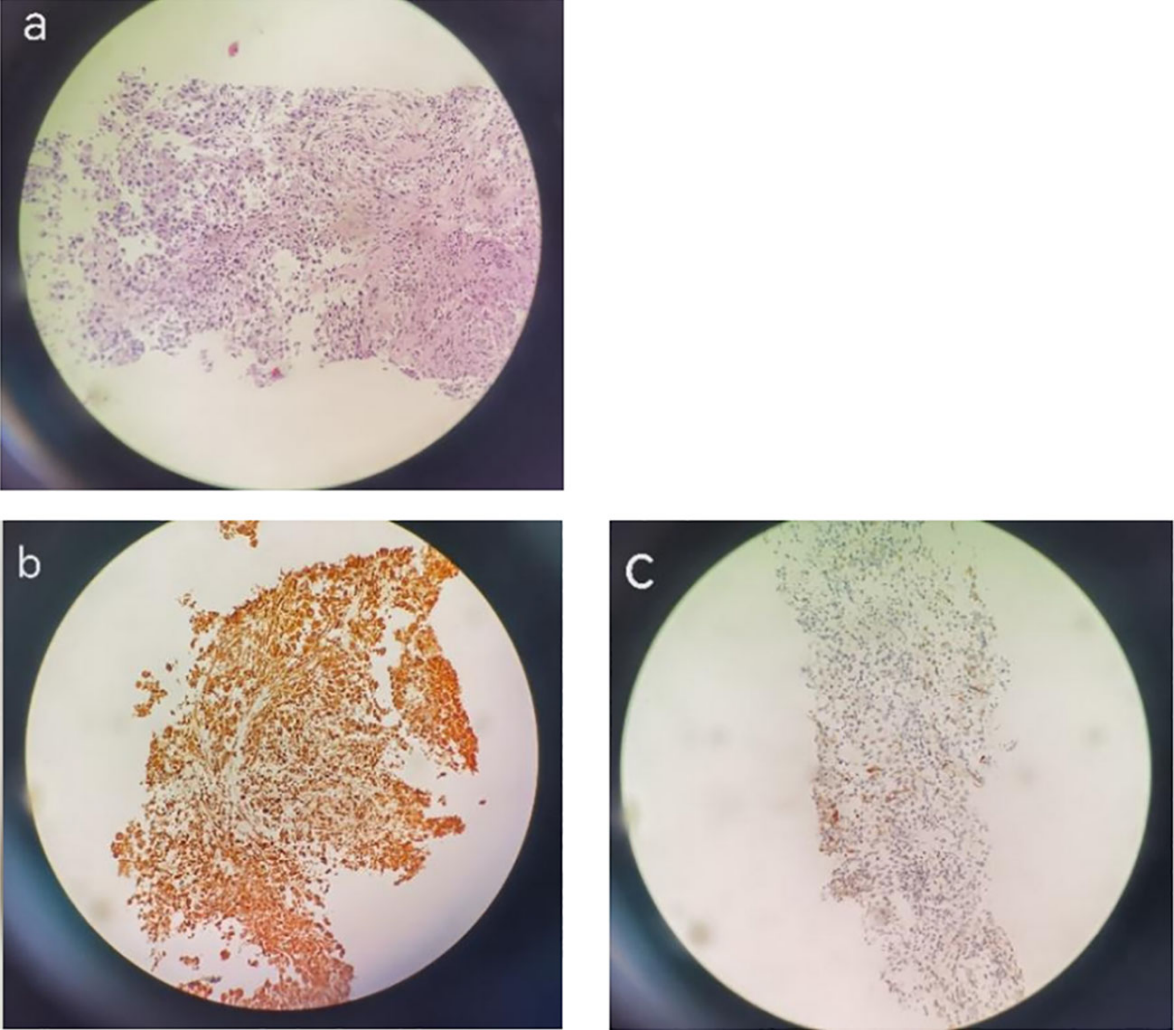
H&E staining. IHC images showing PSC in a 78-year-old man. H&E staining images (magnification: ×200) showing pulmonary sarcomatoid carcinoma **(A)** and IHC images showing Vimentin diffuse positivity (+) **(B)**, CK8(+), CK18 (+) **(C)**.

On 22 December 2020, a CT-guided puncture biopsy was conducted to provide further clarification on the diagnosis of the mass located in the right upper lobe. The biopsy results confirmed the presence of a high-grade malignant tumor. Immunohistochemistry (IHC) analysis revealed negative staining for CK5/6, CK7, Napsin A, P40, CK, TTF-1, S-100, and SMA. However, positive staining was observed for CK8/18, vimentin, and PAS (**Figure 3**). Based on these findings, the patient was diagnosed with stage IIIA primary squamous cell carcinoma (PSC) of the right lung. Genetic testing revealed the presence of the ALK-intergenic (A19: intergenic) rearrangement gene, BUBIB-ALK (B1: A20) rearrangement gene, a missense mutation of KRAS 2 exon p.G12C, TP53 mutation, a TMB of 11.96 mutations per megabase (Mb), MSS status, and PD-L1 expression with a CPS of 3% and a TPS of 2%.

The patient commenced first-line treatment with ALK-TKI (alectinib) targeted therapy in January 2021. Regrettably, after only 1 month, the patient expressed experiencing enlargement of the right neck lymph nodes. Based on the neck ultrasound findings, multiple lymph nodes were observed to be enlarged in the lateral aspect of the right cervical region, suggesting the presence of metastasis. A lung CT scan revealed a slight reduction in size of the right lung mass compared to previous imaging. However, the metastasis in the right hilar and mediastinal lymph nodes appeared slightly larger than before.

The patient received second-line chemotherapy with albumin-bound paclitaxel and cisplatin. Following four cycles of chemotherapy, the size of the mass in the right upper lobe measured 3.5 cm × 2.8 cm on chest CT. The size of the mediastinal lymph nodes increased to 6 cm. The right hilar mass decreased in size compared to previous measurements, and there were no abnormal elevations in tumor markers (**Figure 2**). However, there was an approximately 20% increase in the size of the tumor in the right upper lobe, indicating disease progression recurrence.

**Figure 4:**
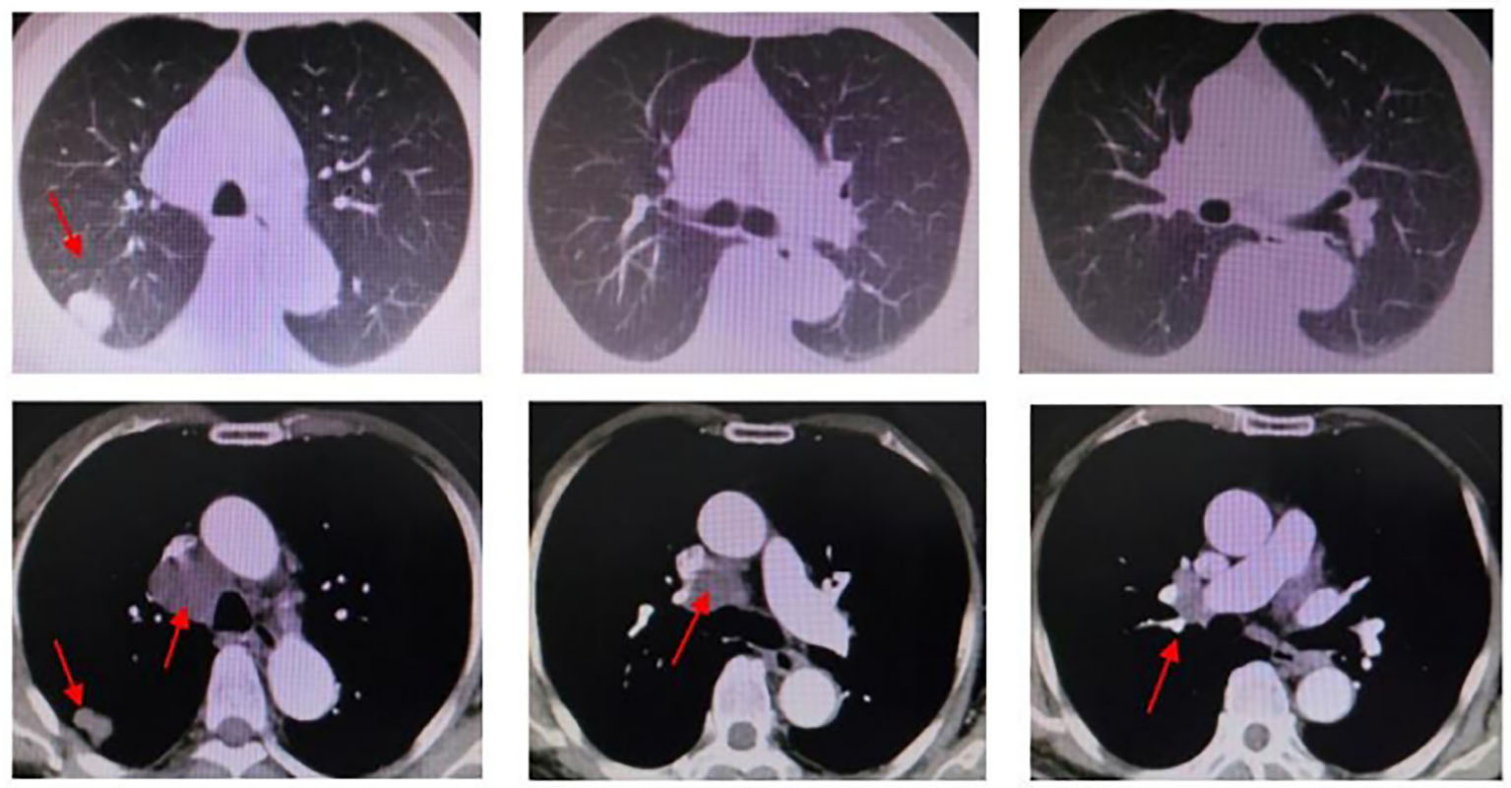
Chest CT (2022.06.27): right upper lobe and right hilar region masses, similar to the previous one. Mediastinal and right hilar lymph nodes metastasis; the larger one is approximately 3.9 * 4.1cm in diameter.

**Figure 5:**
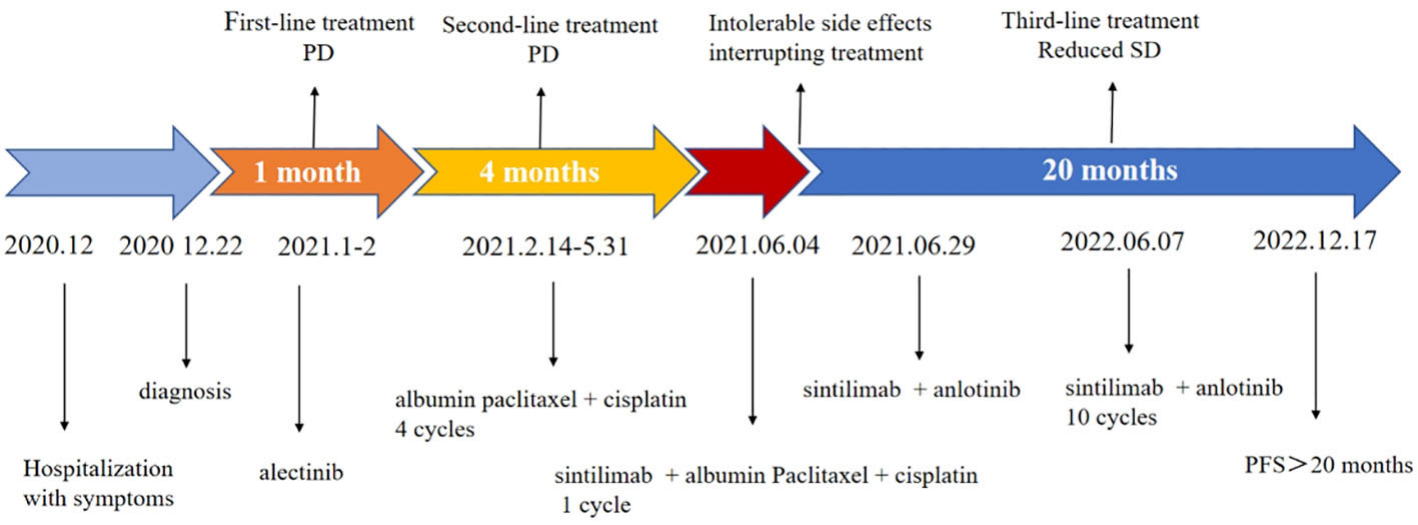
Patient treatment timeline.

In response to disease progression, the treatment regimen was modified to include sintilimab in combination with albumin-bound paclitaxel (sintilimab 200 mg + albumin-bound paclitaxel 200 mg). However, after just one cycle of therapy, the patient experienced grade III weakness and had difficulty tolerating the toxic side effects of chemotherapy. Considering the favorable safety profile of anlotinib, the treatment regimen was modified to sintilimab in combination with anlotinib, starting from 29 June 2021. The evaluation of treatment efficacy showed stable disease (SD) after two cycles of therapy. After six cycles, CT revealed a reduction in the dimensions of the tumor to 2.8 cm × 1.8 cm, which was located in the right upper lobe and right hilar region. Additionally, the metastasis of the mediastinal and right hilar lymph nodes decreased to 4.0 cm × 3.9 cm in diameter. Both showed improvement compared to previous assessments, with a decrease in tumor size, classified as SD. By 7 June 2022, a total of 10 cycles of treatment with the combination of sintilimab and anlotinib were administered. CT scans revealed that the masses in the right upper lobe and right hilar region remained unchanged from the previous findings, while the metastasis of the mediastinal and right hilar lymph nodes still measured 3.9 cm × 4.1 cm in diameter (**Figure 4**). On the ultrasound examination conducted on 4 August 2022, the size of the right supraclavicular metastasis was measured as 0.4 cm × 0.6 cm, which showed a significant reduction compared to the previous measurement. The efficacy evaluation indicated SD. The treatment timeline for the patient is presented in **Figure 5**.

During the administration of the combination therapy of sintilimab and anlotinib, the patient experienced suboptimal glycemic control and increased urinary protein excretion (++-+++). However, these issues were effectively managed symptomatically and did not interfere with the patient’s ongoing treatment. Furthermore, no adverse effects (AEs) related to the combined anti-angiogenic targeted therapy and immunotherapy were observed, including hand-foot syndrome, rash, and hypertension, as well as hepatic, renal, cardiopulmonary, neurological, and thyroid toxicities. The patient achieved stable disease control for a duration of more than 20 months and is still undergoing long-term follow-up.

## Discussion

PSC is a rare subgroup of NSCLC, with poorly differentiated and highly aggressive tumors. Clinical management primarily follows the treatment approach for driver mutation-negative NSCLC, which involves platinum-based chemotherapy. Several studies have indicated that PSC exhibits a high mutation rate, with multigene mutations associated with higher mortality compared to single-gene mutations (10, 11). In accordance with the WHO (2015) classification of lung tumors, it is recommended to test for EGFR mutations and ALK gene fusions in all resection specimens, small biopsies, and cytology specimens containing adenocarcinoma components of PSC. This testing helps determine prognosis, select targeted therapies, and predict treatment efficacy (3).

This case report presents a patient with a polygenic mutation, characterized by a BUBIB-ALK gene rearrangement, KRAS 2 p.G12C missense mutation, and TP53 mutation. Limited evidence exists regarding the use of ALK-TKI in patients with PSC who have both KRAS mutations and ALK rearrangements. However, a previous case report showed a partial response to ALK-TKI in ALK-rearranged PSC patients, which was sustained at 3 months (18). Furthermore, prior to the accelerated FDA marketing approval of Sotorasib (AMG510) on 29 May 2021, KRAS mutations had long been considered to have no targeted drug options. After comprehensive assessment, we chose alectinib (ALK-TKI) as the primary treatment option. Alectinib was approved in November 2017 for the primary treatment of ALK-positive metastatic NSCLC patients. A phase III clinical trial showed that alectinib effectively reduced the risk of disease progression or mortality by 57% and significantly extended the DFS of patients by 34.8 months (19). Furthermore, the safety profile of alectinib was found to be superior to crizotinib. Therefore, alectinib was chosen as the initial therapeutic option. Regrettably, the malignancy demonstrated rapid progression within 1 month.

The underlying mechanism behind the rapid development of resistance to ALK-TKI in this case report remains unclear. For ALK inhibitor resistant patients, it is necessary to consider the relevant testing of ALK acquired resistance mutations. However, due to economic reasons, further biopsy and genetic testing were not conducted to further elucidate the mechanisms behind adverse treatment reactions. Patients with different ALK fusion types have heterogeneous therapeutic responses to ALK-TKI (20). The patient in this case had a rare form of ALK fusion-BUB1B-ALK rearrangement. Unfortunately, we did not find any literature on BUB1B-ALK rearrangement. There is no evidence that ALK inhibition is effective for these rearrangements, and the treatment is mainly carried out with reference to common ALK fusion types. In addition, the classification level of the mutation may also be related to the occurrence of ALK resistance. However, due to the limitations of the research population and differences in the mechanisms of action of different drugs, the results of different studies may vary. Moreover, research on the relationship between the abundance of the mutations and efficacy is still in the exploratory stage. A case study revealed that patients who had both KRAS mutations and ALK rearrangements experienced rapid disease progression despite treatment with crizotinib (21). This observation is believed to be attributed to the coexistence of KRAS and TP53 mutations. KRAS, an enzyme with intrinsic GTPase activity, is frequently mutated in PSC. Studies have indicated that patients with KRAS mutations exhibit reduced responsiveness to TKI therapy (22). Moreover, KRAS mutations in PSC have been associated with a poor OS, indicating that the mutation status of KRAS may serve as a prognostic indicator for PSC. Additionally, TP53 mutations have been identified as a poor prognostic factor for PFS and OS. These mutations have also been associated with the development of multiple ALK resistance mechanisms (23). In patients with TP53 mutations and co-existing EGFR or ALK alterations, there is a diminished response to TKIs. This may be due to increased genomic instability caused by abnormal TP53 function (24). The treatment response observed in this case aligns with existing literature, highlighting that both KRAS and TP53 mutations can confer resistance to ALK TKIs.

Currently, there is no standard treatment for advanced PSC. The current therapeutic approach primarily involves chemotherapy regimens used in patients with NSCLC. In this particular case, after the unsuccessful response to alectinib treatment, the patient chose to use a combination of albumin-bound paclitaxel and cisplatin chemotherapy. Regrettably, the primary lung lesions showed insufficient control after only 3 months of treatment. Subsequently, we implemented a combination therapy approach that included immunotherapy (sintilimab) along with chemotherapy. However, the patient experienced significant intolerance to chemotherapy, which required discontinuation of the treatment. In this case, the patient highlighted the limited efficacy of platinum-based chemotherapy. Additionally, chemotherapy led to significant adverse effects, such as nausea, vomiting, and fatigue, which greatly impacted the patient’s overall well-being. Therefore, novel therapeutic approaches are imperative in order to address this issue.

Vascular invasion is a prominent characteristic and prognostic indicator of poor outcomes in PSC (25). Therefore, anti-angiogenic therapy may offer potential benefits for patients with PSC, who are known to have a higher susceptibility to vascular invasion. Anlotinib is a potent multi-targeted small molecule tyrosine kinase inhibitor that exerts its effects by inhibiting angiogenesis and suppressing the activation of VEGFR2, PDGFRβ, and FGFR1, as well as their shared downstream ERK signaling pathway. In addition, anlotinib enhances immune cell infiltration and modulates the composition of immune cells within tumor tissues by normalizing blood vessels and improving tumor blood perfusion, thereby alleviating the immunosuppressive state in the tumor microenvironment. Consequently, the combination of anlotinib with immunotherapy has the potential to synergistically inhibit tumor progression and enhance patient prognosis (26). In the phase III study ALTER0303, subgroup analysis demonstrated that patients with positive driver gene mutations could still derive significant benefits from anlotinib treatment. This included improved OS and PFS in patients with advanced NSCLC who had received at least two prior lines of treatment (27). Based on these findings, the Chinese Society of Clinical Oncology (CSCO) guidelines have recommended anlotinib as the standard third-line treatment for NSCLC.

A genomic and immunological spectrum analysis conducted on Chinese patients with lung sarcomatoid carcinoma found that the median TMB of pure PSC was 8.6 mutations per megabase (mut/Mb) (10). TMB is defined as the total number of mutations in the DNA of cancer cells, usually expressed as the number of mutations per megabase (mut/Mb) (28). A TMB ≥ 10 mut/Mb is classified as high TMB (TMB-H), which has been associated with a favorable response to immunotherapy (29). The KEYNOTE-158 study demonstrated that the overall response rate in patients with TMB-H was 30.3%, whereas it was only 6.7% in patients without TMB-H. This study represents the first FDA approval of TMB as a biomarker for guiding treatment decisions in patients (13). The findings indicate a high mutation rate and overall mutation load in PSC, which can elicit robust immune responses. Consequently, immunotherapy has shown improved efficacy in these patients, suggesting that ICIs may confer survival benefits (10). This observation is further supported by the patient’s elevated TMB expression (11.96 mut/Mb) and positive response to immunosuppression.

Sintilimab is a highly selective human monoclonal antibody that specifically targets the PD-1 receptor on immune cells. It was developed in China by independent research groups and acts by interfering with PD-1-mediated anti-tumor immune signal transduction. Sintilimab has been approved as a first-line treatment for advanced NSCLC. Retrospective studies have demonstrated that patients with NSCLC who did not respond to systemic treatment showed positive outcomes when treated with a combination of sintilimab and anlotinib (30). Based on the documented anti-tumor efficacy of ICIs, as well as the favorable survival outcomes observed with the combination of anlotinib and PD-1/L1 inhibitors, we chose the therapeutic approach involving sintilimab in conjunction with anlotinib. This treatment regimen has been clinically validated to provide significant benefits to patients. Notably, the patient’s disease has remained stable for over 20 months, and ongoing treatment and follow-up are being conducted as part of this program.

Based on the premise that immunotherapy could still be beneficial in cases with low PD-L1 expression, we attempted to investigate the correlation between PD-L1 expression, TMB, and gene mutations in relation to therapeutic options and prognosis for this patient. Although evidence suggests that co-mutations of KRAS and TP53 are associated with unfavorable clinical outcomes in NSCLC, patients with modified TP53 and KRAS mutations are more likely to derive benefits from anti-PD-1/PD-L1 immunotherapy compared to without these mutations. This benefit may be attributed to a significant increase in the expression of PD-L1 and TMB (23). In retrospective studies involving patients with lung adenocarcinoma, it has been confirmed that the presence of dual mutations in KRAS and TP53 is associated with elevated levels of TMB, inflammatory markers, and immune checkpoint effector molecules. This indicates that these patients may show increased sensitivity to PD-L1 antibody therapy (31).

The patient presented with concurrent BUBIB-ALK rearrangement and KRAS G12C mutation. The patient has undergone multiple lines of therapy, including ALK-TKI targeted therapy, platinum-based chemotherapy, anlotinib, and sintilimab. Remarkably, the patient has achieved favorable local control, with the PFS exceeding 20 months, without experiencing any significant drug-related adverse events. This case highlights that, in advanced PSC patients, the combination of targeted therapy with anti-angiogenic agents and ICIs may led to promising treatment outcomes. This combination offers a well-tolerated option for maintenance treatment, provided that adverse events are effectively managed.

## Conclusion

In conclusion, we demonstrated the efficacy of a combination therapy using sintilimab (a PD-1 inhibitor) and anlotinib (a small molecule anti-angiogenic agents) to treat advanced PSC patients with BUBIB-ALK rearrangement and KRAS G12C mutation, which had shown resistance to ALK-TKI treatment. This therapeutic approach resulted in a prolonged PFS and improved quality of life. These findings provided compelling evidence for the effectiveness of ICIs and anlotinib in the treatment of PSC. However, no literature pertaining to BUB1B-ALK rearrangement was identified, possibly due to its rare occurrence. The case appears to be an exceptional instance and holds promise for shedding light on the therapeutic approach for patients with similar rearrangements. Consequently, it is imperative to conduct more stratified studies and to refine the management strategies for individuals with ALK-positive alterations, as this bears significant clinical relevance. Further research is necessary to gain a better understanding of the gene mutation and its mechanism of action in PSC. Anti-angiogenic therapy and immunotherapy may offer potential benefits for patients, as opposed to traditional radiotherapy and chemotherapy. However, more robust and comprehensive clinical trials or studies are needed to validate the efficacy of these treatments.

## Data availability statement

The original contributions presented in the study are included in the article/supplementary material. Further inquiries can be directed to the corresponding authors.

## Ethics statement

Written informed consent was obtained from the individual(s) for the publication of any potentially identifiable images or data included in this article.

## Author contributions

CZ: Conceptualization, Data curation, Writing – original draft. JL: Conceptualization, Data curation, Supervision, Writing – original draft. TQ: Data curation, Resources, Visualization, Writing – original draft. NH: Conceptualization, Formal analysis, Writing – original draft.
